# It is not good to talk: conversation has a fixed interference cost on attention regardless of difficulty

**DOI:** 10.1186/s41235-018-0124-5

**Published:** 2018-08-22

**Authors:** Melina A. Kunar, Louise Cole, Angeline Cox, Jessica Ocampo

**Affiliations:** Department of Psychology, The University of Warwick, Coventry, CV4 7AL UK

## Abstract

It is well-documented that telephone conversations lead to impaired driving performance. Kunar et al. (Psychon Bull Rev 15:1135–1140, 2008) showed that this deficit was, in part, due to a dual-task cost of conversation on sustained visual attention. Using a multiple object tracking (MOT) task they found that the act of conversing on a hands-free telephone resulted in slower response times and increased errors compared to when participants performed the MOT task alone. The current study investigates whether the dual-task impairment of conversation on sustained attention is affected by conversation difficulty or task difficulty, and whether there was a dual-task deficit on attention when participants overheard half a conversation. Experiment 1 manipulated conversation difficulty by asking participants to discuss either easy questions or difficult questions. The results showed that there was no difference in the dual-task cost depending on conversation difficulty. Experiment 2 showed a similar dual-task deficit of attention in both an easy and a difficult visual search task. Experiments 3 and 4 showed that in contrast to work using a dot tracking and choice reaction time task (Emberson et al., Psychol Sci 21:1383–1388, 2010), there was little deficit on MOT performance of hearing half a conversation, provided people heard the conversations in their native language. The results are discussed in terms of a resource-depleted account of attentional resources showing a fixed conversational-interference cost on attention.

## Significance

The impairments of talking on a mobile phone while driving have been well-documented with people showing slower response times and making more errors when conversing on a phone compared to when they are silent (e.g. Strayer & Johnston, 2001). There are many components of driving - one of which is the need to pay attention to the road. Previous work has investigated whether attentional tasks on their own are impaired when people engage in the act of conversation (Kunar, Carter, Cohen, & Horowitz, 2008). The results showed that even in a single-attention task, speed and accuracy are impaired when the participant converses compared to when they do not. However, it is not yet known whether different types of conversation affect attention differently, with more difficult conversations leading to greater impairments. Neither is it known whether the effect of conversing causes a fixed impairment in attention regardless of task difficulty or whether conversation impairs difficult tasks to a greater degree than it does easier ones. We investigated this and showed that conversation has a fixed impairment on attention regardless of conversation or task difficulty. Finally, we showed that there is little impairment in a sustained attention task of overhearing half a conversation. Given that mobile phone use is on the rise (Pickrell & Ye, 2009) our results are significant in demonstrating that regardless of how easy or difficult people believe a task to be, there is a measurable fixed impairment to attention tasks when engaged in a conversation.

## Background

As we go about our daily lives we often perform several tasks simultaneously. Sometimes these two tasks are simple and can be performed together easily (e.g., humming while walking). Other times performing two tasks concurrently is much harder and leads to a performance detriment in either or both tasks (e.g., driving while talking on a mobile phone, Strayer & Johnston, 2001). These performance costs are known as dual-task deficits and have been studied extensively in the laboratory (e.g. Allen, McGeorge, Pearson, & Milne, 2006; Allport, Antonis, & Reynolds, 1972; Fougnie & Marois, 2006; Kunar, Shapiro, & Humphreys, 2006; Pashler & O’Brien, 1993).

Recent research into dual-task deficits have investigated the cost of talking on a mobile telephone while driving (e.g., Breim & Hedman, 1995; Strayer & Drews, 2007; Strayer, Drews, & Crouch, 2006; Strayer & Johnston, 2001). It has consistently been found that telephone conversation leads to impaired driving performance. For example, Strayer et al. (2006) compared participants who were under the influence of alcohol to those who were talking on a mobile phone. Both groups of participants showed significant impairments in driving performance. Furthermore, Strayer and Drews (2007) found people failed to recall objects that they directly viewed more often, showing greater inattentional blindness, when they were involved in a conversation while driving compared to when they were not. Importantly, these effects were not due to motor conflicts of holding a telephone as the dual-task deficit was still observed in conditions where the conversation took place on a hands-free device (Strayer & Johnston, 2001). Clearly, there is a great cost of talking while driving. However, the reason for this cost is not yet fully understood.

One explanation for this conversational impairment is that the act of conversing interferes with attentional processes. Driving in itself is a task that involves multiple components. For example, in order to successfully manipulate a car, the driver has to check mirrors, steer and manipulate brake/gas pedals all while paying visual attention to the road conditions. Given the various sub-tasks, it is unclear which parts of driving are affected by the act of conversation. Strayer and Johnston (2001) suggested that as the impairment was not affected by peripheral motor issues, such as holding the phone, then the cost was attentional in its nature. Kunar et al. (2008) directly tested this claim by using a multiple object tracking (MOT) task designed to measure sustained attention alone (Horowitz et al., 2007; Pylyshyn & Storm, 1988; Wolfe, Place, & Horowitz, 2007), without the other added confounds of driving.

In the MOT task, participants were shown eight disks on a computer screen and asked to track a subset of these disks. Evidence shows that people are usually able to successfully track around four out of eight disks (Cavanagh & Alvarez, 2005, although see Alvarez & Franconeri, 2007, who showed that this tracking capacity varied with disk speed and Watson & Kunar, 2012, who determined capacity limits depend on stimulus’ properties). Kunar et al. (2008) asked participants to perform an MOT task on its own and while they were talking on a hands-free telephone and showed that people were slower and less accurate at responding whilst having a conversation. The data clearly demonstrate that even attentional tasks on their own had a dual-task cost when participants were engaged in conversation. We investigate the nature of this effect further in the current paper by examining the influence of differing cognitive demands and conversation type. In particular, we investigate how conversational difficulty and task difficulty affect the dual-task cost of conversation on visual attention. Furthermore, we examined whether there was also a dual-task cost of hearing half a conversation on visual attention. To give an overview of the results we found a decrease in attentional task performance when participants were engaged in a conversation, but minimal disruption on attentional task performance when they listened to a conversation.

Bergen, Medeiros-Ward, Wheeler, Drews, and Strayer (2013) have investigated whether the deficit of conversation occurs due to domain-general or domain-specific reasons. They suggested that the reason for the dual-task cost can be broadly split into two categories. The first category (domain-general) refers to the idea that people have a limited number of attentional resources with which to complete all the tasks they need to do. If the tasks being performed concurrently use up more resources than there are available, then performance of one or both of the tasks suffers (e.g., Kahneman, 1973). This theory stipulates that there need not be any overlap in these tasks in terms of modality (e.g., visual or auditory) or processing codes (e.g., manual or verbal) for dual-task interference to occur; instead there is a generalised processing cost leading to a domain-general deficit. The second category (domain-specific) suggests that interference from concurrently performed tasks only occurs when they share common resources. Therefore, there will be greater interference between two visual tasks compared to a visual and an auditory/speech task. This theory is in line with the multiple resource theory proposed by Wickens (1984, 2002). Bergen et al. (2013) concluded that different attentional theories cover different components of driving. For example, braking reaction times (RTs) in a simulated driving test showed a domain-general interference effect, whereas following distance showed a domain-specific interference effect.

Regardless of which theory accounts for the dual-task interference effect, both the domain-general and domain-specific theories state that the magnitude of the interference effect should be influenced by conversation and/or task difficulty. For example, previous research has robustly shown that increasing task difficulty can lead to impairments in RTs and error rates (e.g., Kunar, Thomas, & Watson, 2017; Kunar & Watson, 2011, 2014; Wolfe, Oliva, Horowitz, Butcher, & Bompas, 2002). That is, the more difficult a task is the more resources it will use, leaving fewer available for a second task, leading to greater dual-task impairments (e.g., Hitch & Baddeley, 1976; Kahneman, 1973; Sullivan, 1976). We call this a *difficulty-dependent* account of attentional resources. In relation to sustained attention this would predict that if conversation were to become more difficult, performance on the MOT task would suffer relative to when conversation was easier. Conversely, it has also been suggested that the dual-task deficit occurs because people become so involved in the conversation that they fail to respond to the competing task (Wickens, 2002; see also Helleberg & Wickens, 2003). A strong version of this account would suggest an “all or nothing” effect where the mere act of conversing would lead to a fixed delay in responding and would not be modulated by conversation difficulty. We call this the *resource-depleted* account, where the dual-task interference of conversation on attention leads to a fixed depletion of attentional resources, regardless of conversation and/or task difficulty.

Previous research has looked at the effect of conversation difficulty on simulated driving tasks. Breim and Hedman (1995) had participants converse in either an easy or difficult conversation while completing a simulated driving task. In the easy condition participants were asked to engage in naturalistic conversation with the experimenter. In the difficult condition participants had to correctly identify if a sentence was logical or not while also recalling the first words of the previous four sentences. The results found that performance in the simulated driving task was worse when participants were involved in the difficult conversation versus the easy conversation. Furthermore, it has been found that performing a more difficult word generation task impaired simulated driving and MOT performance compared to performing an easier speech shadowing task (see Strayer & Johnston, 2001 and Kunar et al., 2008, respectively). Evidence from these studies are in favour of a difficulty-dependent account, stating that the more demanding a task, the more cognitive resources it uses, leaving fewer attentional resources available for subsequent tasks.

Please note that although the results of the aforementioned studies are informative the types of conversational tasks involved across conditions are very different (e.g., a conversation versus a working memory task in the study by Breim & Hedman, 1995 and a repeating a word versus a cognitive word-generation task in Strayer & Johnston, 2001 and Kunar et al., 2008). Furthermore, working memory tasks, shadowing tasks and word generation tasks are not often utilised in everyday speech. Experiment 1 in the present study addresses these issues directly by having both the easy conversation and difficult conversation conditions based on a series of questions. Not only are the answering of questions more common in everyday speech, they allow the type of conversation to remain constant across the experiment while allowing manipulation of task difficulty. To preview the results, when the type of conversation was controlled for by asking questions in both conditions, a similar dual-task deficit on MOT performance occurred with both the difficult and the easy conversations, in line with a resource-depleted account of attentional resources.

Experiment 2 manipulated the difficulty of the attentional task. Here we changed the task to a visual search task, so that the attentional resources needed to complete the task could be easily manipulated (Treisman & Gelade, 1980). Participants completed two visual search tasks whilst conversing: an easy single-feature task and a more difficult spatial-configuration task. In the easy task, participants were instructed to look for a red target, T, among green distracter Ls. As the target was different to the distracters by a unique feature (colour) we would expect the RTs in this task to remain stable over set size and produce efficient search slopes of around 0 ms/item (Treisman & Gelade, 1980; Watson & Kunar, 2010; Wolfe & Horowitz, 2004). In this type of search task the target “pops out” without the need for attention (Treisman & Gelade, 1980). In the difficult spatial-configuration task participants were asked to look for a white target, T, among white distracter Ls. As the target and distracters were similar to each other in colour and line orientation, participants had to actively search the display, which produced inefficient search slopes, where RTs increased with set size (e.g., Duncan & Humphreys, 1989; Kunar & Humphreys, 2006; Kunar, Rich, & Wolfe, 2010). According to a difficulty-dependent account, as the single-feature task can be completed pre-attentively (e.g., Treisman & Gelade, 1980), there should be little deficit to this task of having a conversation. However, as the difficult spatial-configuration task requires greater attentional resources there should be a dual-task cost of conversation in this task. Conversely, the resource-depleted account predicts a dual-task cost independent of task difficulty, therefore, according to this account we would expect a dual-task cost in both the easy and difficult search tasks. The results showed that the act of conversing led to a dual-task deficit in *both* visual search tasks, consistent with a resource-depleted account.

The second part of this paper investigated whether hearing part of a telephone conversation also had similar detrimental effects on sustained attention. Emberson, Lupyan, Goldstein, and Spivey (2010) previously found that overhearing half a conversation was more attentionally demanding than hearing a whole conversation. In their study, they had participants perform a dot-tracking task and a choice RT task where participants had to respond to target letters and ignore distracter letters (Emberson et al., 2010). Participants performed both these tasks while listening to (a) half a conversation (a “halfalogue”) comprising speech from one person’s part of a conversation, (b) a dialogue of both people’s parts of a conversation and (c) a monologue where they heard one person’s recap of a conversation. The results showed that performance was impaired in the halfalogue condition compared to the dialogue and monologue conditions. From these results, Emberson et al. (2010) suggested that overhearing a conversation would also be disruptive to driving and other attentional tasks. We investigate this here by comparing the disruption of hearing half a conversation on a sustained visual attention task to that of having a conversation. If a dual-task cost was observed when people heard part of a conversation then this would have implications for the dangers of overhearing passenger conversations when driving. In experiment 3 we had participants perform an MOT task in three conditions: (1) when they were listening to a halfalogue, (2) when they were involved in a conversation and (3) in silence. Experiment 4 more closely replicated the conditions used by Emberson et al. (2010) by comparing MOT performance when participants overheard half a conversation to when they overheard a dialogue between two people. The results showed that provided that the conversation was in a person’s native language, overhearing half a conversation did little to disrupt sustained attention. Taken together the results of experiments 1–4 showed that, although *listening* to a conversation did little to affect attention, *engaging* in a conversation had a large detrimental effect on attentional performance, regardless of conversational or task difficulty. We discuss the applied and theoretical implications of these data further in “General discussion”.

## Experiment 1

### Method

#### Participants

Thirty participants (mean age = 22.1 years, 18/30 female) took part in the experiment. A power analysis using G* Power (Faul, Erdfelder, Lang, & Buchner, 2007) with the effect size taken from the original experiment reported in Kunar et al., 2008 (experiment 1, which showed a significant difference in MOT performance between conversation and no conversation conditions, with an effect size = 0.7, α = .05, two-tailed) found that using this number of participants would achieve a power of 0.96 in each of the experiments presented subsequently. All participants had normal or corrected-to-normal vision. Ethical approval for all experiments was obtained from the Department of Psychology, University of Warwick ethics board. All participants gave informed consent before taking part in the experiment.

#### Stimuli and procedure

Displays were generated and responses recorded by custom written computer programmes running on a PC. Stimuli were eight dark grey disks subtending 1° of visual angle at a viewing distance of 57 cm. The background was a uniform light grey.

There were three MOT conditions: a no conversation condition, a difficult conversation condition and an easy conversation condition. For the no conversation condition, at the beginning of each trial participants were presented with a fixation dot at the centre of the computer screen for 1 s. They were then presented with four dark grey disks (the “distracter disks”) and four yellow disks (the “target disks”). These remained on the screen for 1 s before the target disks changed their colour to dark grey (matching the distracter disks). These remained on the screen for 500 ms, at which point all eight disks began to move at a constant speed of 6.7°/s in a randomly assigned and unpredictable direction (making sure that the disks never occluded one another). After 3 s the disks stopped moving and one of the eight disks turned red. Participants were asked to respond to whether the red disk was a target or not by pressing one of two keys (“m” if the red disk was the target and “z” if it was the distracter). The red disk was a target in 50 % of trials. Participants were asked to respond as quickly but as accurately as possible and error rates and RTs were recorded.

**Fig. 1:**
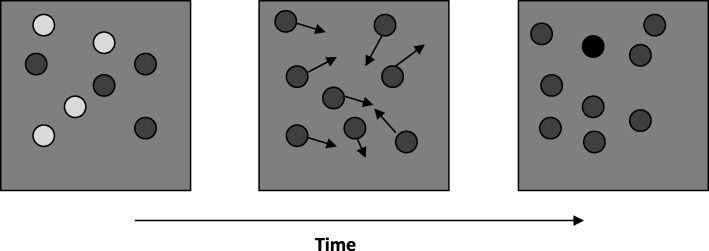
Example display of experiment 1. The light grey disks in the first frame were yellow in the experiment proper and the black disk in the last frame was red

The difficult conversation and the easy conversation condition were similar to the no conversation condition except that in these conditions participants engaged in a conversation with the experimenter while doing the MOT tasks. The conversation continued over multiple MOT trials to last the length of the entire condition. Topics of conversation were taken from “The Book of Questions” (Stock, 1985). Fifteen additional questions, that had been previously judged to be either difficult or easy in a pilot study, were also used. In the difficult conversation condition, throughout the MOT task participants were asked to respond to a series of questions that were difficult to answer, for example, “if you were able to live to the age of 90 and retain either the body or the mind of a 30-year-old for the last 60 years of your life which would you want?”. In the easy conversation participants were asked a series of questions throughout the MOT condition that were easy to answer, for example, “when is your birthday?” Each set of questions was independently coded by 15 volunteers who rated each question for difficulty on a 7 point Likert scale (1 = very easy; 7 = very difficult). The results showed that the easy set of questions were rated to be easier than the difficult set of questions (2.9 vs 3.9, respectively, *t* (14) = 5.4, *p* < 0.01, *d* = 4.128). Furthermore, the time taken for the volunteers to respond to the first ten questions from the easy set was quicker than when asked to respond to the first ten questions from the difficult set (64.9 s vs 193.3 s, respectively, *t* (14) = 10.6, *p* < 0.01, *d* = 3.418) again indicating that the easy set of questions were easier to answer than the difficult set.^1^ For each condition there were 50 trials per participant. Participants were given a short practice block before each condition and the order of conditions in this experiment (and all subsequent experiments) was counterbalanced. Example displays can be seen in Fig. 1.

### Results and discussion

**Fig. 2:**
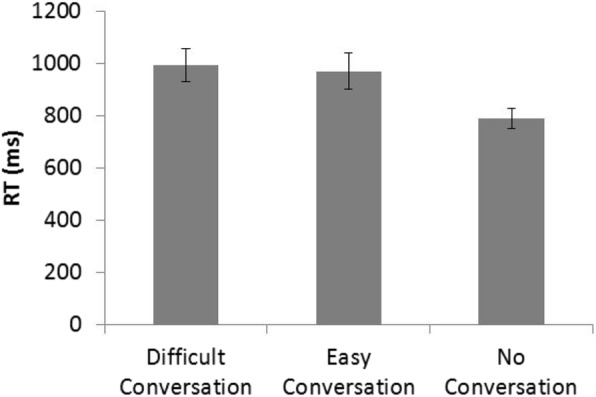
Reaction times (RTs) for each condition in experiment 1. Error bars represent the standard error

**Fig. 3:**
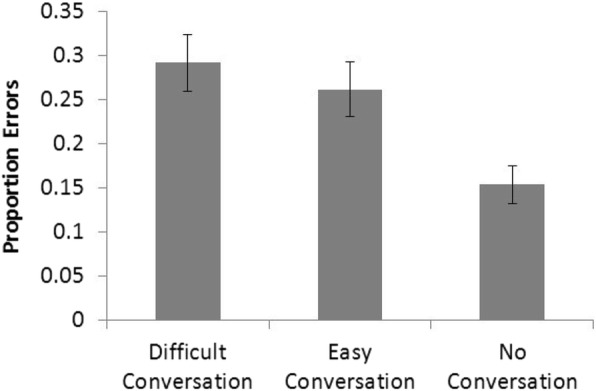
The proportion of errors for each condition in experiment 1. Error bars represent the standard error

Trials with RTs less than 200 ms or greater than 4000 ms were removed as outliers (2.5% of the data). Figure 2 shows the mean correct RTs and Fig. 3 shows the error rates for each condition. Within-participants analysis of variance (ANOVA) for mean correct RTs showed there to be a main effect of condition, *F*(2, 58) = 17.1, *p* < 0.01, *η*_*p*_^2^ = 0.371. Planned *t* tests showed a significant difference between the no conversation and difficult conversation condition, *t* (29) = 5.1, *p* < 0.01, *d* = 0.872, where RTs were slower in the difficult conversation condition compared to the no conversation condition, and a significant difference between the no conversation and easy conversation condition, *t* (29) = 4.5, *p* < 0.01, *d* = 0.718, where RTs were slower in the easy conversation condition compared to the no conversation condition. There was no difference in RTs between the easy conversation and difficult conversation condition, *t* (29) < 1, *d* = 0.073.

Examining the error rates, within-participants ANOVA showed there to be a main effect of condition, *F* (2, 58) = 21.7, *p* < 0.01, *η*_*p*_^2^ = 0.428. Planned *t* tests showed a significant difference between the no conversation and difficult conversation condition, *t* (29) = 5.5, *p* < 0.01, *d* = 1.10, where more errors were made in the difficult conversation condition compared to the no conversation condition, and a significant difference between the no conversation and easy conversation condition, *t* (29) = 4.7, *p* < 0.01, *d* = 0.870, where more errors were made in the easy conversation condition compared to the no conversation condition. There was a trend for fewer errors to occur in the easy conversation condition than in the difficult conversation condition, *t* (23) = 1.7, *p* = 0.09, *d* = 0.217.

The results were clear. Performance in the MOT task was impaired both in terms of speed and accuracy when people were simultaneously having a conversation. This occurred regardless of whether the content of the conversation was designed to have participants answer relatively difficult questions or simple ones. Comparing both RTs and error rates for the easy and difficult conversation conditions we see no modulation of the dual-task deficit in RTs and only a slight increase in error rates in the difficult condition. The data are hard to reconcile with a difficulty-dependent account of attentional resources, which would predict that the dual-task deficit would increase with conversation difficulty. Instead the data are in favour of a resource-depleted account where having a conversation leads to a fixed delay in attentional response regardless of difficulty.

One could argue that having a difficult conversation could be considered more engaging than having an easier conversation. Perhaps this level of engagement led to an improvement in RTs (as participants might be less prone to having their “mind wander” if they were actively engaged), which would counter the disruptive effect of conversation on MOT performance. Although possible, we believe this to be unlikely as Wickens (2002) proposed that it is the *engaging* nature of the conversation that leads to the dual-task deficit. They proposed that when participants were actively engaged in a conversation they “dropped” the secondary task entirely (Wickens et al., 2002). Furthermore, previous research has shown that asking a participant to be actively engaged in search (opposed to passively searching the display) leads to an impairment in attentional performance, rather than an improvement (Smilek, Enns, Eastwood, & Merikle, 2006; see also Lleras & Von Mühlenen, 2004, Olivers & Nieuwenhuis, 2005, Kunar, Watson, Cole, & Cox, 2014, Watson, Brennan, Kingstone, & Enns, 2010 and Kunar, Ariyabandu, & Jami, 2016, who showed that actively giving participants a choice led to an increase in RTs and search efficiency). Therefore, if anything, having participants be more engaged in a conversation would predict an impairment in performance, rather than an improvement. However, this did not occur. Instead the data are easier to reconcile with a resource-depleted account suggesting that having any type of conversation (easy or difficult), leads to a fixed cost in attention.

Experiment 1 showed a dual-task impairment on MOT performance while participants were simultaneously engaged in conversation (see also Kunar et al., 2008). Experiment 2 investigated whether conversation also impaired performance in visual search. This experiment investigated the effect of conversation on two different types of search task: one that produced efficient search (the “easy search” condition) and one that produced inefficient search (the “difficult search” condition). Does the disruption of conversation also extend to these tasks and will task difficulty lead to different dual-task impairments when paired with conversation?

## Experiment 2

### Participants

Thirty participants (mean age = 21.2 years, 21/30 female) took part in the experiment. All had normal or corrected-to-normal vision.

### Stimuli and procedure

**Fig. 4:**
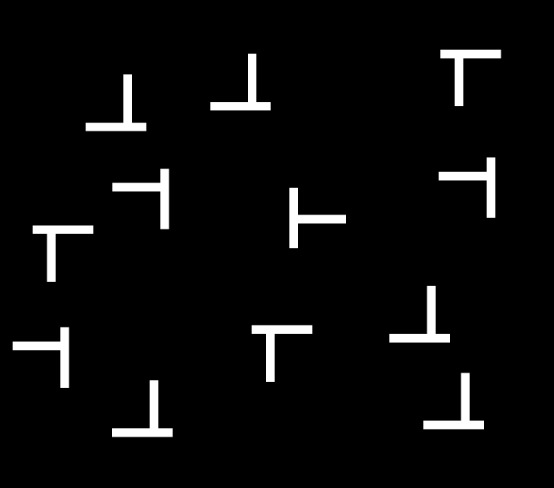
Example display of experiment 2. In the single-feature condition the target T was red and the distracter Ls were green. In the spatial configuration condition all stimuli were white

Displays were generated and responses recorded by custom written computer programs running on a PC. The stimuli were rotated Ts and Ls. Each stimulus had a visual angle of 1.7° × 1.7° at a viewing distance of 57 cm and the vertical lines of the Ls were slightly offset from its horizontal line (see Fig. 4 and Russell & Kunar, 2012, for examples of these stimuli). All stimuli were presented on a black background. On each trial, there were either 4, 8 or 12 stimuli presented. The target was present on every trial.

There were two visual search conditions: the easy search condition and the difficult search condition. In the easy search condition participants were asked to search for the target letter of a red T among distracter letters of green Ls. As the target was defined by a unique feature it would require little search and “pop-out” of the search display (Treisman & Gelade, 1980). In the difficult search condition participants were still asked to search for the letter T; however, in this condition all the stimuli were white. Given that the target had no defining feature and the fact that there was a great deal of similarity between the target and the distracters, these sets of stimuli would result in inefficient search (e.g., Duncan & Humphreys, 1989; Wolfe, Cave, & Franzel, 1989).

To begin each trial, a blank screen appeared for 500 ms and was followed by a central fixation point for 500 ms. The T and L stimuli were then presented randomly within an invisible 6 × 6 matrix. All stimuli were presented randomly in one of four orientations (0°, 90°, 180° or 270°) with equal probability. Participants were asked to indicate the direction in which the stem of the target T was pointing, by pressing “m” if the stem faced right and “z” if the stem faced left. Participants were instructed to respond as quickly but as accurately as possible. If no response was made within 10 s, the trial “timed out” and the next trial automatically started. Following a response or time-out, the blank screen was again displayed before the next fixation point and trial. There were 90 trials in each condition (30 for each set size).

Participants completed each visual search (easy and difficult) twice - once on its own and once while conversing with the experimenter. The conversation was similar to the conversation conditions of experiment 1, except that instead of answering questions participants were asked to engage in a naturalistic conversation with the experimenter whilst completing the visual search task. The conversation topics were not limited; however, the experimenter opened the conversations with topics such as recreational interests, holiday plans and travel preferences. If one topic of conversation naturally ended the researcher started another conversation on a different topic. Conditions were counterbalanced and RTs and error rates were recorded. Participants were asked to respond as quickly but as accurately as possible.

### Results and discussion

**Fig. 5:**
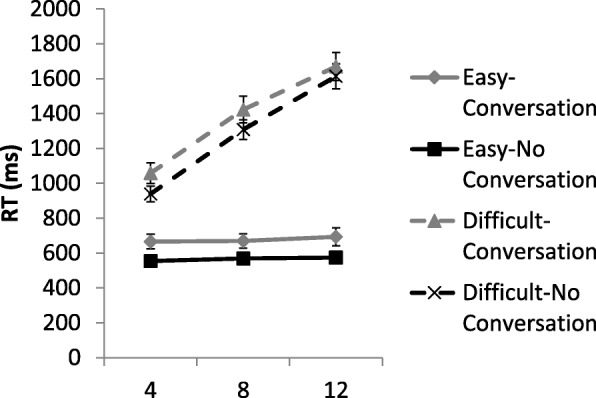
Reaction times (RTs) across set size for each condition in experiment 2. Error bars represent the standard error

**Fig. 6:**
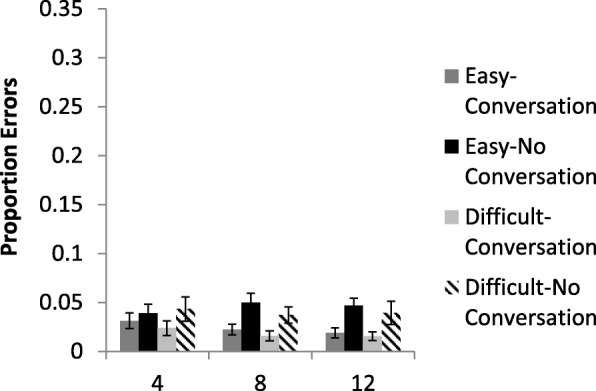
The proportion of errors across set size for each condition in experiment 2. Error bars represent the standard error

Trials with RTs less than 200 ms or greater than 4000 ms were removed as outliers (1.2% of the data). Figure 5 shows the mean correct RTs and Fig. 6 shows the error rates for each condition. Search slopes are shown in Table 1. As expected, search in the single-feature search was efficient (with search slopes close to 0 ms/item), while search in the spatial configuration task was inefficient. Mean correct RTs were entered into 2 × 2 × 3 ANOVA with main effects of visual search type (easy vs difficult), conversation (conversation vs no conversation) and set size (4, 8 or 12). As expected there was a main effect of visual search type, *F* (1, 29) = 290.8, *p* < 0.01, *η*_*p*_^*2*^ = 0.909, where RTs for the difficult search condition were slower than those for the easy search condition. There was a main effect of conversation, *F* (1, 29) = 12.7, *p* < 0.01, *η*_*p*_^2^ = 0.305, where RTs in the conversation condition were slower than in the no conversation condition and there was a main effect of set size, *F* (2, 58) = 358.4, *p* < 0.01, *η*_*p*_^2^ = 0.925, where RTs increased with set size. The visual search type × set size interaction was also significant, *F* (2, 58) = 224.0, *p* < 0.01, *η*_*p*_^2^ = 0.885, reflecting that RTs increased with set size more in the difficult search task than in the easy search task, as expected. Importantly, none of the other interactions involving conversation were significant (all *F* values <1.5).

**Table 1:** Search slopes for each condition in experiment 2

Condition	Search slope (ms/item)
Easy-conversation	3.4
Easy-no conversation	2.6
Difficult-conversation	76.4
Difficult-no conversation	84.4

Examining the error rates, 2 × 2 × 3 ANOVA showed there to be a main effect of conversation, *F* (1, 29) = 10.5, *p* < 0.01, *η*_*p*_^2^ = 0.266, where surprisingly there were more errors in the no conversation than in the conversation condition (4.3% vs 2.1%, respectively). There was no main effect of visual search type, *F* (1, 29) = 1.3, *p* = 0.27, *η*_*p*_^2^ = 0.042, nor was there a main effect of set size, *F* < 1, *η*_*p*_^2^ = 0.017. None of the interactions were significant (all *F* values <1.2, *ps* > 0.33).

**Fig. 7:**
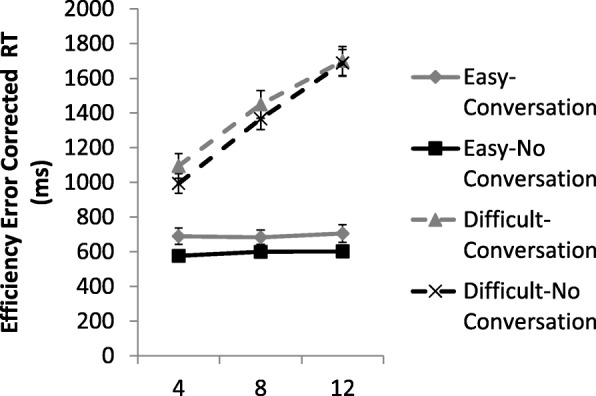
Efficiency across set size for each condition in experiment 2. Error bars represent the standard error. RT, reaction time

Even though the error rates were low throughout the experiment, as the RTs and error rates showed opposite patterns of results in terms of the effect of conversation, the RTs and error rates were combined to produce one overall measure of Efficiency (RT corrected for error rates). This was calculated by dividing the RT by the proportion of correct responses and is a method to determine the overall pattern of results, taking into account both RTs and error rates (Allen, Humphreys, & Matthews, 2008; Townsend & Ashby, 1983). Figure 7 shows the measure of efficiency for all conditions. The 2 × 2 × 3 ANOVA showed there to be a main effect of visual search type, *F* (1, 29) = 230.2, *p* < 0.01, *η*_*p*_^2^ = 0.888, where efficiency was worse in the difficult search condition than in the easy search condition. There was a main effect of conversation, *F* (1, 29) = 7.4, *p* < 0.05, *η*_*p*_^2^ = 0.203, where efficiency was worse in the conversation condition than in the no conversation condition and there was a main effect of set size, *F* (2, 58) = 313.6, *p* < 0.01, *η*_*p*_^2^ = 0.915, where efficiency decreased with increasing set size. As expected, the visual search type × set size interaction was also significant, *F* (2, 58) = 217.3, *p* < 0.01, *η*_*p*_^2^ = 0.882, reflecting that efficiency decreased with set size more in the difficult search task than in the easy search task. Importantly, none of the other interactions involving conversation were significant (all *F* values <2.1, *ps* > 0.14).

The overall pattern of results showed a dual-task deficit of conversation in both visual search tasks. A difficulty-dependent account would have predicted that as detection of the target in the single-feature task was pre-attentive (Treisman & Gelade, 1980), it would need fewer attentional resources and thus be exempt from the deficit of conversation (or at least show less of a dual-task impairment compared to the difficult condition). However, this was not the case. Instead the results concurred with a resource-depleted account, showing that regardless of task difficulty there was a fixed dual-task cost of having a conversation. Please note that the interference of conversation in both conditions only affected the RTs and did not affect the search slopes. This suggests that the cost of conversation was due to a delay in the central bottleneck of processing rather than interfering with the mechanism behind the search process. We discuss this further in “General Discussion”.

The results from experiment 1 and 2 showed a significant dual-task cost of conversing on visual attention, regardless of conversation or task difficulty. Experiment 3 examined whether a similar dual-task deficit was observed when participants only heard half of a conversation (mimicking conditions where people were exposed to one side of a mobile phone conversation). Emberson et al. (2010) identified attentional impairment when people overheard half a conversation. However, one could argue that the cognitive act of conversing would lead to a greater depletion of attentional resources, as it is a more difficult and complex task than simply listening to a conversation (Kunar et al., 2008; Strayer & Johnston, 2001). In this case, we would expect a greater dual-task deficit to occur in the conversation condition compared to overhearing half a conversation.

## Experiment 3

### Participants

Thirty participants (mean age = 24.2 years, 24/30 female) took part in the experiment. All had normal or corrected-to-normal vision.

### Stimuli and procedure

The stimuli and procedure were similar to that of experiment 1 except that there were three conditions: a no conversation condition, a conversation condition and a half conversation condition. The no conversation and conversation conditions were the same as those in experiment 2. In the half conversation condition, participants heard the researcher engage in a halfalogue whilst completing the MOT task. In this condition the researcher sat in the same room as the participant and engaged in a scripted half conversation, maintaining all of the non-verbal and non-vocal aspects of the conversation such as pauses, sighs, laughter etc. The script for the half conversation condition was created by transcribing half of an actual telephone conversation between friends.

### Results and discussion

**Fig. 8:**
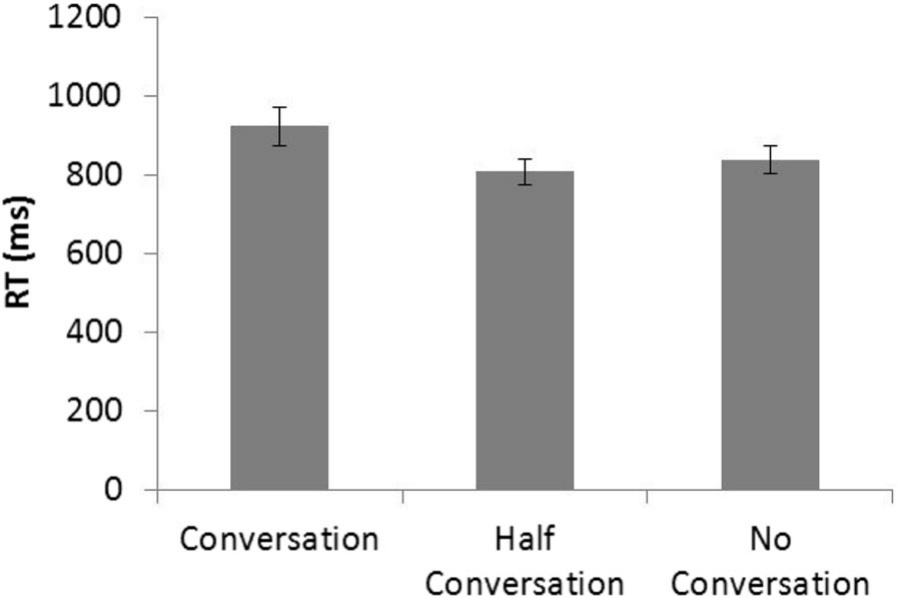
Reaction times (RTs) for each condition in experiment 3. Error bars represent the standard error

**Fig. 9:**
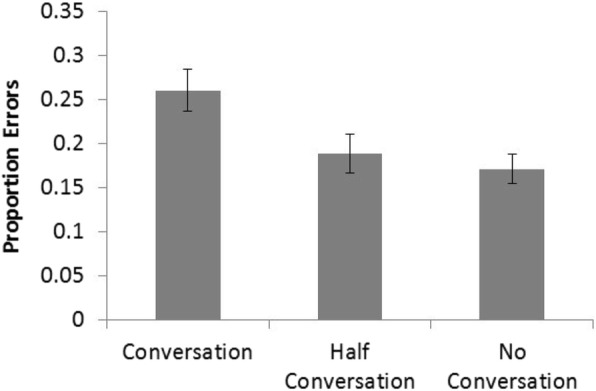
The proportion of errors for each condition in experiment 3. Error bars represent the standard error

Trials with RTs less than 200 ms or greater than 4000 ms were removed as outliers (0.4% of the data). Figure 8 shows the mean correct RTs and Fig. 9 shows the error rates for each condition. Within-participants ANOVA for mean correct RTs showed there to be a main effect of condition, *F* (2, 58) = 7.5, *p* < 0.01, *η*_*p*_^2^ = 0.206. Planned *t* tests showed a significant difference between the half conversation and conversation conditions, *t* (29) = 3.3, *p* < 0.01, *d* = 0.498, where RTs in the conversation condition were slower than in the half conversation condition. RTs were also slower in the conversation condition compared to the no conversation condition, *t* (29) = 2.5, *p* = 0.02, *d* = 0.356. There was no significant difference, however, between the half conversation and no conversation condition, *t* (29) = 1.5, *p* = 0.16, *d* = 0.158.

Examining the error rates, within-participants ANOVA showed there to be a main effect of condition, *F* (2, 58) = 15.8, *p* < 0.01, *η*_*p*_^2^ = 0.353. Planned *t* tests showed a significant difference between the half conversation and conversation conditions, *t* (29) = 3.7, *p* < 0.01, *d* = 0.560, where more errors were made in the conversation condition compared to the half conversation condition. There was also a significant difference between the conversation and no conversation condition, *t* (29) = 5.2, *p* < 0.01, *d* = 0.805, where more errors were made in the conversation condition compared to the no conversation condition. However, there was no significant difference between the half conversation and no conversation condition, *t* (29) = 1.3, *p* = 0.21, *d* = 0.171.

The results again showed a dual task detriment on MOT performance, in terms of both speed and accuracy, while participants were engaged in conversation. However, examining the results of the half conversation condition showed there to be no dual-task detriment in speed or accuracy in comparison to the no conversation condition. Clearly listening to half of a conversation did little to impair performance in a sustained attentional task. These results are inconsistent with those of Emberson et al. (2010) who found that overhearing half a conversation was disruptive to a dot tracking and choice RT task. Note that in their experiments, results from the half conversation condition were also compared directly to a dialogue condition where participants overheard a full conversation, rather than participants taking part in a conversation. We replicated these conditions more closely in experiment 4, by examining the effect of overhearing a dialogue compared to overhearing half a conversation when performing an MOT task.

## Experiment 4

### Participants

Thirty participants (mean age = 23.3 years and 21/30 female) took part in the experiment. All had normal or corrected-to-normal vision.

### Stimuli and procedure

The stimuli and procedure were similar to that of experiment 3 except that there were three conditions involved: a no conversation condition, a dialogue condition and a half conversation condition. The no conversation and half conversation conditions were the same as that in experiment 3, except that the halfalogue was recorded and played to participants through headphones (the same halfalogue was used in this condition as that in experiment 3). In the dialogue condition, participants heard a full dialogue of a conversation between two people. Both the halfalogue and dialogue conversations were recorded via an iPhone and played through headphones using Microsoft Windows media software. The use of pre-recorded conversations more closely replicated the methodology of Emberson et al. (2010) who also used pre-recorded messages. To ensure participants were listening to each conversation they were told that they were going to be asked questions about each conversation after the dialogue and half conversation conditions. Directly after each of these conditions participants answered three multiple-choice questions on the content of the conversation. The questions were asked and the answers were recorded by the experimenter.

### Results and discussion

**Fig. 10:**
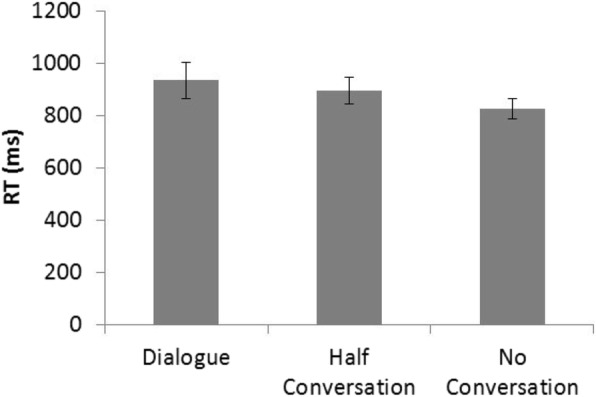
Reaction times (RTs) for each condition in experiment 4. Error bars represent the standard error

**Fig. 11:**
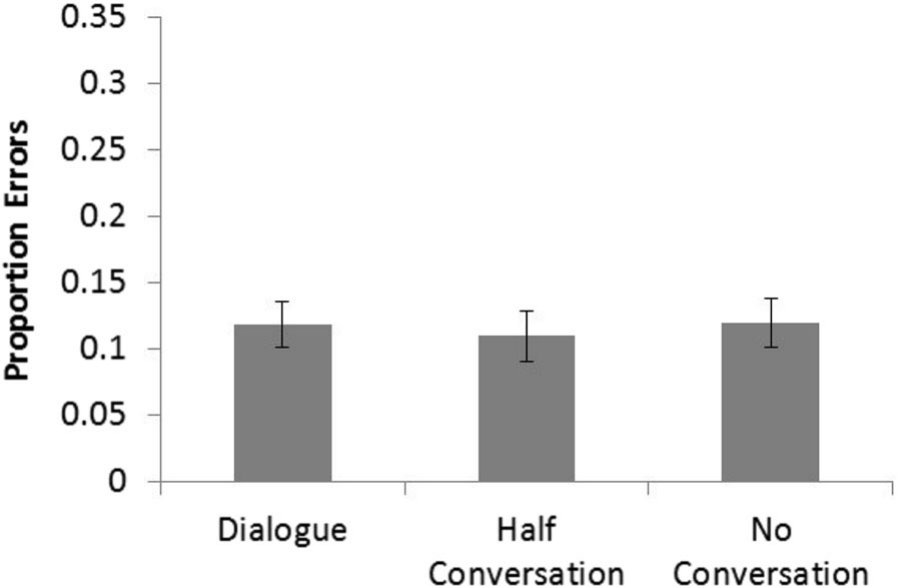
The proportion of errors for each condition in experiment 4. Error bars represent the standard error

One participant was excluded from analysis as they only answered 50% of the multiple-choice questions correctly.^2^ Data from the remaining participants showed a mean accuracy rate of 95% and were analysed further. Trials with RTs less than 200 ms or greater than 4000 ms were removed as outliers (0.5% of the data). Figure 10 shows the mean correct RTs and Fig. 11 shows the error rates for each condition. Within-participants ANOVA for mean correct RTs in the MOT tasks showed there to be a main effect of condition, *F* (2, 56) = 3.7, *p* = 0.03, *η*_p_^2^ = 0.118. Planned *t* tests showed a significant difference between the dialogue conversation and the no conversation conditions, *t* (28) = 2.7, *p* = 0.01, *d* = 0.362, where RTs in the dialogue condition were slower than in the no conversation condition. RTs were also marginally slower in the half conversation condition compared to the no conversation condition, *t* (28) = 1.8, *p* = 0.09, *d* = 0.285. There was no significant difference, however, between the half conversation and dialogue condition, *t* (28) = 0.9, *p* = 0.35, *d* = 0.120. Examining the error rates, within-participants ANOVA showed there to be no main effect of condition, *F* (2, 56) = 0.4, *p* = 0.66, *η*_*p*_^2^ = 0.015.

The results are mixed. Although the error rates concur with those of the previous experiment, the RT results conflict with those in experiment 3. In experiment 3 the results showed that there was no effect of overhearing half a conversation compared to the no conversation condition on RTs. However, in experiment 4, there was a trend for RTs to be slower when overhearing half a conversation compared to hearing no conversation. Furthermore, RTs in the dialogue condition were slower to those in the no conversation condition, which is different to the findings of Emberson et al. (2010). There are two possible reasons for the discrepancy in results. The first could be that in experiment 4 people were asked questions about the conversation or half conversations they heard. However, no questions were asked about the half conversation in experiment 3. It could be that the act of listening to the conversation in order to answer questions led to reduced cognitive resources for performing the MOT task. Although theoretically plausible we do not believe this to be the case as previous research has robustly shown that listening tasks do not interfere with attentional performance - even if participants had to answer questions about what they had heard (Kunar et al., 2008; Strayer & Johnston, 2001).

The second explanation could be that in experiment 4 there was a higher proportion of participants whose native language was different to that of the conversation (there were 7%^3^ vs 48% of non-native speakers in experiments 3 and 4, respectively). This meant that comprehension of the conversations may have been more difficult for these participants leading to more cognitive resources being used.^4^ This in turn would have led to a reduction of resources available to perform the MOT task in the halfalogue and dialogue conditions and an overall increase in RTs. There is some merit to this hypothesis if we break down the results across participants who categorised themselves as native speakers versus those that categorised themselves as non-native speakers in experiment 4 (14 vs 13 participants, respectively - 2 participants declined to categorise themselves and were excluded from further analysis). Looking at participants who reported themselves as native speakers we see that there was no difference in RTs between any of the conditions, *F* (2, 26) = 0.9, *p* = 0.462, *η*_*p*_^2^ = 0.063 (829 ms, 771 ms and 795 ms for the dialogue, half and no conversation condition, respectively). However, comparing participants who categorised themselves as non-native speakers we see that there was a difference in RTs across condition, *F* (2, 26) = 4.35, *p* = 0.02, *η*_*p*_^2^ = 0.266 (1064 ms, 1032 ms and 869 ms, for the dialogue, half and no conversation condition, respectively). For these participants, RTs were slower in the dialogue condition than the no conversation condition, *t* (12) = 2.5, *p* = 0.03, *d* = 0.538, and slower in the half conversation condition than the no conversation condition, *t* (12) = 2.6, *p* = 0.02, *d* = 0.611. There was no difference in RTs between the dialogue and half conversation condition, *t* (12) = 0.45, *p* = 0.7, *d* = 0.082. The data suggest that for non-native speakers there was a cost to RTs of overhearing either the whole or part of a conversation on MOT performance. However, in agreement with experiment 3, when the conversation was spoken in a person’s native language there was little cost of overhearing either the whole or part of a conversation.

## General discussion

Previous work has shown a dual-task cost of having people engage in a sustained attentional task while also having a conversation (Kunar et al., 2008). The present data show the robustness of this dual-task detriment of conversation across a variety of conditions. Experiment 1 showed that regardless of whether the conversation content was relatively easy or relatively difficult, both conversation types impaired MOT performance in comparison to a condition where there was no conversation. Experiment 2 showed that conversing also impaired performance in an easy, efficient visual search task as well as in an inefficient search task. Experiments 3 and 4 showed that, unlike previous research using dot tracking and choice RT tasks, there was no dual-task impairment on MOT of overhearing half a conversation on error rates. Furthermore, there was little dual-task impairment on RTs between overhearing half a conversation and having no conversation, provided that the participants listened to a conversation in their native language.

### Conversation and difficulty

Our results have important implications for the nature of conversation on performance in attentional tasks. Experiment 1 showed that both easy and difficult conversations have similar impact on attentional demands. Wickens (2002), see also Helleberg & Wickens, (2002) suggested that the reason conversation disrupted performance on a secondary task is that the act of conversing is so engaging that participants drop the secondary task altogether. A strong version of this claim would suggest that any conversation, regardless of difficulty would lead to impaired performance of a secondary task. Our results are in favour of this claim supporting a resource-depleted account, whereby the engagement of conversing, regardless of difficulty, impedes performance in attentional tasks.

One could argue that we did not see any difference between the easy and difficult conditions due to a lack of power. For example, there was a hint of a difference in the error rates between the difficult and easy conditions in experiment 1, where there was a marginal effect (29% vs 26%, respectively). However, as the effect size for this comparison was small, we do not believe there is truth in a strong version of this account. Furthermore, any differences between the difficult and easy conditions were not observed in the reaction times, nor in experiment 2 (which also directly manipulated task difficulty). Last, given that the power level was above 0.95 in all experiments, a lack of power in these experiments seems unlikely. Instead the experiments demonstrate that both the easy and difficult conversation conditions showed a marked impairment compared to when no conversation was taking place. Generalising this back to a driving situation, the results clearly indicate that having a conversation, regardless of difficulty level, resulted in a depletion of attention that could have serious consequences on the road.

Furthermore, experiment 2 showed that conversation even had a detrimental effect on tasks that are considered to require very little in the way of attentional resources (e.g., the single-feature task). These data are challenging to explain by a difficulty-dependent account which would suggest that as the single-feature task needs very few attentional resources, it should be performed well under dual-task conditions with little impairment. However, this was not the case. As a dual-task cost was witnessed in both the inefficient and efficient search tasks the results instead point to a resource-depleted account, where there was an overall cost of conversing, regardless of task difficulty. When looking at performance in these visual search tasks we can glean some insight into the nature of this conversational interference. According to experiment 2 we see that the deficit of conversation led to an overall RT cost rather than a change to the search slopes. This would suggest that the nature of the conversational interference was due to the psychological refractory period (PRP) as there was an overall additive delay in responding, rather than interference with the actual search mechanisms (see Levy, Pashler, & Boer, 2006, who also found a dual-task PRP effect in a simulated driving task). The PRP effect occurs when two signals, requiring response are presented in close temporal proximity to each other (Welford, 1952). It is found that response to the second signal is delayed until the response to a first signal has been completed (e.g., Pashler, 1994; Welford, 1952). In the case of our work, people would be delayed in responding to the visual search task whilst they were engaged in conversation. Please note that this delay could be dangerous if we extrapolate it back to driving conditions. For example, when travelling at the speed of 60 miles/hour a 110 ms delay in response^5^ could lead to travelling an extra distance of 9.7 ft before braking. This is over half the length of a standard-sized car.

### Attentional impact of hearing half a conversation

Previous research has found that listening to half a conversation (a halfalogue) is more disruptive than hearing a whole conversation or a monologue on a manual dot-tracking task and a choice RT task (Emberson et al., 2010). This is thought to be because the unpredictable nature of hearing half a conversation disrupts these simple attentional tasks. These results are important as there could be consequences for tasks like driving if overhearing a passenger conversation affects sustained attention. However, our results show that this was not the case: although there was strong evidence of an impairment in performance when taking part in a conversation, there was only minimal disruption to MOT performance when participants listened to half a conversation, providing the conversation was in their native language.^6^ The difference in *listening* to a conversation and *engaging* in a conversation on attention is most likely due to the attentional resources each task requires. Although experiments 1 and 2 showed that the difficulty of the conversation did not affect MOT performance, previous research has shown that having a conversation is a more complex task than just listening to speech (Kunar et al., 2008; Strayer & Johnston, 2001). For example, Kunar et al. (2008) found that the cognitive act of generating speech in a word generation task interfered more with attentional processes compared to just listening to a narrative or shadowing a word. Our current research shows that the cognitive act of having a conversation, regardless of difficulty, depletes attentional resources more so than overhearing half of other people’s conversations.

Overall, our findings do not concur with those of Emberson et al. (2010), who found disruption of overhearing half a conversation compared to overhearing a whole conversation or no conversation. There are several potential reasons for this difference. One reason could be that the dot tracking and choice RT tasks used by Emberson et al. (2010) were more sensitive to smaller amounts of distraction compared to the MOT task. For example, there could be circumstances in the MOT task when, although participants were distracted, they would still be able to track the targets with some degree of success (for example, if the target was far away from other distractors),^7^ meaning that the MOT task was less susceptible to distraction. Alternatively, the difference in results could have been due to the perceptual load of the attentional task. Participants had to track four disks in the MOT task compared to one disk in the dot-tracking task. This difference meant that the MOT task had a higher perceptual load than the task used by Emberson et al. (2010). Previous research has shown that the perceptual load of a task effects how many attentional resources there are available for processing other stimuli (e.g. Lavie, 1995). If the perceptual load of the task is high (as in the MOT task) then there are fewer attentional resources to process the halfalogue and there will be less interference from it. In contrast if the perceptual load is lower (the dot tracking and choice RT task) then there will be extra attentional resources available to process the halfalogue leading to greater interference. It is up to future work to investigate this further, however the present data give us insight into the boundaries of mobile phone use in sustained attentional tasks, like that of driving. Although talking on a telephone has negative consequences there is no detriment of overhearing half a passenger’s conversation.

### Domain-general versus domain-specific accounts

The current data also address the issue of what theory is responsible for the dual-task attentional deficit observed while conversing. Bergen et al. (2013) found, when looking at braking RTs, a domain-general theory best accounted for their results; however, when they looked at following distance they found some evidence for a domain-specific account. Our results can give insight into which theory is responsible for the dual-task deficit of conversation on attention. Across experiments a strong and robust dual-task deficit was observed. This occurred even though there was minimal overlap between the modalities of each task: the MOT task required visual resources while the conversation required auditory and speech resources. As a domain-specific account would only predict interference between tasks that shared common resources (e.g. modalities, processing codes etc.) then it is difficult to reconcile these data with a strong version of this account. Instead, we conclude that a domain-general account is theoretically more likely to be responsible for the deficit of conversation on attention.

### Implications for attention in road safety and other tasks

The experiments described show that the act of conversing has detrimental effects on sustained attention. This has implications for a number of daily tasks including the act of driving. Lochner and Trick (2014) showed that it is possible to track multiple vehicles while driving (although tracking interferes with both headway and lane positioning) and that people were faster and more accurate at detecting changes to vehicles they were tracking. This included detection of changes in the sudden onset of brake lights. Given that our research has shown that conversation interferes with tracking performance, it is likely that conversation will also interfere with picking up important changes made to tracked vehicles, potentially leading to dangerous situations (i.e. responding too late to an onset of a sudden brake light). Paying attention to your visual surroundings is imperative for safely navigating the outside world not only while driving in a vehicle, but as a pedestrian too. Furthermore, sustained attention is crucial for a number of other important socio-economic and everyday tasks (e.g., air traffic control, operating machinery, baggage screening, searching for cancers on mammograms, looking after a child). As the use of mobile phones has been on the rise (Glassbrenner, 2004; Pickrell & Ye, 2009) it is important to know the risks to attention of engaging in a conversation. These data add to the growing literature in the field showing that even the most simplistic of conversations can have profound interference effects on tasks that require attention.

## Abbreviations

ANOVA: Analysis of variance
MOT: Multiple object tracking
PRP: Psychological refractory period
RT: Reaction time

## Footnotes

1 As there were more questions in the “easy” set than the “difficult” set we could not make a comparison of RTs to respond to *all* questions in each condition. Therefore, we chose to measure RTs to the first ten questions in each condition as an indicator of difficulty.

2 Note that the pattern of data remained the same if this participant was included in data analysis.

3 Unfortunately, we do not have data for 2 of the participants and so have excluded them from this statistic: among the remaining 28 participants, 2 were non-native speakers in experiment 3.

4 Note that all participants self-reported having good levels of English (the conversations were all presented in English) and answered the majority of the questions about the conversations correctly. However, it may be that more cognitive resources were needed for those who spoke English as a second language rather than those who spoke it as a first language.

5 This is the average RT delay in the single feature condition of experiment 2, when participants were having a conversation

6 Please note that the analysis in experiment 4 comparing native and non-native speakers was post hoc and therefore should be handled with caution. However, despite this the data from experiment 3 clearly showed there was little disruption of listening to half a conversation on MOT performance.

7 We thank Trafton Drew for this suggestion.
